# Rational mutagenesis to support structure-based drug design: MAPKAP kinase 2 as a case study

**DOI:** 10.1186/1472-6807-9-16

**Published:** 2009-03-18

**Authors:** Maria A Argiriadi, Silvino Sousa, David Banach, Douglas Marcotte, Tao Xiang, Medha J Tomlinson, Megan Demers, Christopher Harris, Silvia Kwak, Jennifer Hardman, Margaret Pietras, Lisa Quinn, Jennifer DiMauro, Baofu Ni, John Mankovich, David W Borhani, Robert V Talanian, Ramkrishna Sadhukhan

**Affiliations:** 1 Department of Biochemistry, Abbott Laboratories, Worcester, MA USA; 2 Department of Molecular Pharmacology, Abbott Laboratories, Worcester, MA USA; 3 Department of Molecular Cell Biology, Abbott Laboratories, Worcester, MA USA; 4 Present address : Department of Physical Biochemistry, Biogen Idec, Cambridge, MA USA; 5 Present address : Department of Process Sciences, Abbott Laboratories, Worcester, MA USA; 6 Present address : 119 North Swain Street, Raleigh, NC 27601, USA; 7 Present address : Protein Sciences Department, Genomics Institute of the Novartis Research Foundation, San Diego, CA USA; 8 Present address : Department of Biologics, Abbott Laboratories, Worcester, MA USA; 9 Present address : D. E. Shaw Research, New York, NY USA

## Abstract

**Background:**

Structure-based drug design (SBDD) can provide valuable guidance to drug discovery programs. Robust construct design and expression, protein purification and characterization, protein crystallization, and high-resolution diffraction are all needed for rapid, iterative inhibitor design. We describe here robust methods to support SBDD on an oral anti-cytokine drug target, human MAPKAP kinase 2 (MK2). Our goal was to obtain useful diffraction data with a large number of chemically diverse lead compounds. Although MK2 structures and structural methods have been reported previously, reproducibility was low and improved methods were needed.

**Results:**

Our construct design strategy had four tactics: *N*- and *C*-terminal variations; entropy-reducing surface mutations; activation loop deletions; and pseudoactivation mutations. Generic, high-throughput methods for cloning and expression were coupled with automated liquid dispensing for the rapid testing of crystallization conditions with minimal sample requirements. Initial results led to development of a novel, customized robotic crystallization screen that yielded MK2/inhibitor complex crystals under many conditions in seven crystal forms. In all, 44 MK2 constructs were generated, ~500 crystals were tested for diffraction, and ~30 structures were determined, delivering high-impact structural data to support our MK2 drug design effort.

**Conclusion:**

Key lessons included setting reasonable criteria for construct performance and prioritization, a willingness to design and use customized crystallization screens, and, crucially, initiation of high-throughput construct exploration very early in the drug discovery process.

## Background

Structure-based drug design (SBDD) can be an effective contributor to the identification and optimization of drug candidates by providing a structural rationale for the design of improved compounds. Protein crystallization, structure determination, and the rapid determination of multiple protein/ligand complexes can be expensive and time-consuming. Major variables include protein construct design, mutations, and post-translational modifications, the nature of protein impurities (chemical or conformational), the choice of ligands or even proteins for co-crystallization, and the crystallization conditions themselves. These variables represent an enormous matrix of experimental possibilities that is difficult or impossible to explore systematically. Despite these challenges, the availability of structural information at the preliminary stages of a drug discovery program is critical to maximize impact. Therefore, efficient methods developments, techniques and strategies to deliver structures early in a project are clearly needed.

MAPKAP kinase 2 (MK2) plays a key role in the production of pro-inflammatory cytokines such as TNF-α. MK2 is activated by the mitogen-activated protein (MAP) kinase p38 [1-3]. Activated MK2 phosphorylates a number of target proteins in immune cells resulting in cytokine production and cellular proliferation and activation. Mice lacking MK2 are healthy and fertile, but they fail to increase production of pro-inflammatory cytokines such as TNF-α, IL-6, and IFN-γ [4] in response to stimuli such as lipopolysaccharide. MK2 knockout mice are resistant to the development of collagen-induced arthritis, a model for human rheumatoid arthritis [5]. The catalytic activity of MK2 is required to mount the pro-inflammatory response [6]. These and related studies have attracted attention to MK2 as a target for the design of therapeutic treatments for rheumatoid arthritis and other TNF-α-driven diseases.

Although the data supporting MK2 as a promising drug target have been available for nearly ten years, to our knowledge there are no MK2 inhibitors in clinical development. Many companies have initiated MK2 projects, but little success has been reported. Anecdotally, a common problem has been that high-throughput screening for lead MK2 inhibitors has been unproductive. We believe SBDD targeting MK2 could help address this issue. Yet, despite several reports of MK2 crystal structures at moderate (2.7–3.8 Å) resolution [7-10], the routine production of well-diffracting MK2 crystals bound to compounds of diverse structure remains difficult. More robust methods are needed to enable efficient SBDD.

The domain structure of MK2 may contribute to these difficulties [2]. Its proline-rich *N*-terminal domain (residues 1–65) is unique, having no counterpart in other MAP kinases [3]. This domain binds *c*-ABL Src homology 3 domain *in vitro *[11]. The sequence of the kinase domain (66–327) identifies MK2 as a Ser/Thr kinase family member. MK2 exhibits low homology to other Ser/Thr kinases, however, with the exception of the close homologs MK3 and MK4. The regulatory domain at the *C*-terminus (328–400) contains an autoinhibitory α-helix [7,8] followed by nuclear export signal (NES) and nuclear localization signal (NLS) sequences [2,3,12-15]. The NES and NLS are essential for MK2 complex formation with p38 and subsequent translocation to the nucleus. Deletion of the entire MK2 regulatory domain results in a marked increase in catalytic activity [15]. There are three critical phosphorylation sites on MK2: Thr222, Ser272, and Thr334 [16,17]. Phosphorylation at these residues activates MK2 by causing a conformational change in the *C*-terminal regulatory α-helix: on kinase activation, the helix displaces from the kinase surface and thereby allows substrates to bind [7,9,16].

Construct design is known to be a critical factor in producing large quantities of soluble protein and reproducible crystals. For example, it can be difficult to predict precisely domain boundaries and to identify the surface residues of globular proteins, alteration of which might enhance solubility or crystallization. Altering or deleting features such as surface hydrophobicity, post-translational modifications, side-chain flexibility, secondary structural elements, or even entire domains can dramatically modulate protein physical characteristics, especially solubility. Protein solubility also depends on details such as the expression vector, host cell, culture conditions, and protein fusion partner used. To increase crystallization robustness and improve crystal diffraction, we thought such wide-ranging approaches needed to be explored with MK2.

Here we report the optimization of several steps in human MK2 structure determination: rapid and systematic exploration of construct design and expression screening; high-throughput protein purification; and wide screen crystallization with customized factorial grids. Our methods expand upon those of Malawski *et al. *[18], who examined only *N*- and *C*-terminal truncations of the MK2 catalytic domain. We took advantage of the fact that MK2 is one of the few kinases that expresses well in *Escherichia coli*, which facilitates high-throughput construct design and production, to explore the effect of not only truncations but also two kinds of surface mutations and several internal deletions.

Our strategy had four components: First, the *N*- and *C*-termini of the protein were varied. Second, surface-exposed lysine and glutamate residues with high conformational entropy [19] were mutated to drive novel crystallization contacts and thereby enhance crystallization. Third, internal flexible regions were deleted, again to foster novel crystal forms. Fourth, phosphorylation sites [16,17] were altered to provide homogenous MK2 rather than a heterogeneous mixture of unactivated (no phosphorylation) and activated (partial or full phosphorylation) forms.

We implemented a high-throughput, parallel approach to enable construct production, expression, and purification of all mutants within a short time, nearly all of which expressed well and were tested in customized, kinase-specific robotic crystallization screens. The methodological improvements implemented here enabled the screening of 44 MK2 constructs, resulting in seven crystal forms, diffraction testing of ~500 crystals, and high-resolution data collection and structure determination of ~30 MK2/inhibitor complexes.

## Results and discussion

We initiated MK2 crystallographic studies with a construct (Table 1) comprising part of the proline-rich domain, the kinase and *C*-terminal regulatory domains, and a point mutation introduced to abolish kinase activity. MK2(36–401, K93R) disrupts the highly conserved catalytic lysine residue; the catalytically-inactive K93R mutation was described previously [6]. We began with an inactive construct because use of inactive kinases proved critical to our obtaining homogenous protein suitable for crystallography on several earlier projects. It quickly became apparent, however, that these MK2 constructs were not suitable for structural studies, due to both low expression levels and relative insolubility, likely due in part to the proline-rich segment. We switched to a construct that had been used to determine the first reported MK2 crystal structure, MK2(47–400) [7]. Although protein expression and behavior improved, suitable crystals were not forthcoming, despite success in another laboratory. We believed that a new, more robust approach was clearly needed.

**Table 1:** Representative MK2 expression constructs.

**Rationale**	**Constructs**
*N*- and *C*-terminal variations	MK2(36–400, K93R) MK2(41–364) MK2(47–357) MK2(47–366)

Entropy-reducing surface mutations	MK2(41–364, K64A) MK2(41–364, K343A, E344A) MK2(47–366, K84A) MK2(47–366, E88A, K89A)

Activation loop deletions	MK2(41–364, Δ(L220-G238)) MK2(47–366, Δ(L220-G238))

Pseudoactivation mutations	MK2(41–364, T222E) MK2(41–364, T334E) MK2(41–364, T222E, T334E) MK2(47–366, T222E) MK2(47–366, T334E) MK2(47–366, T222E, T334E)

The complete list of MK2 constructs is available in Additional File 1 Table S7.

### Construct Design Strategy

**Figure 1:**
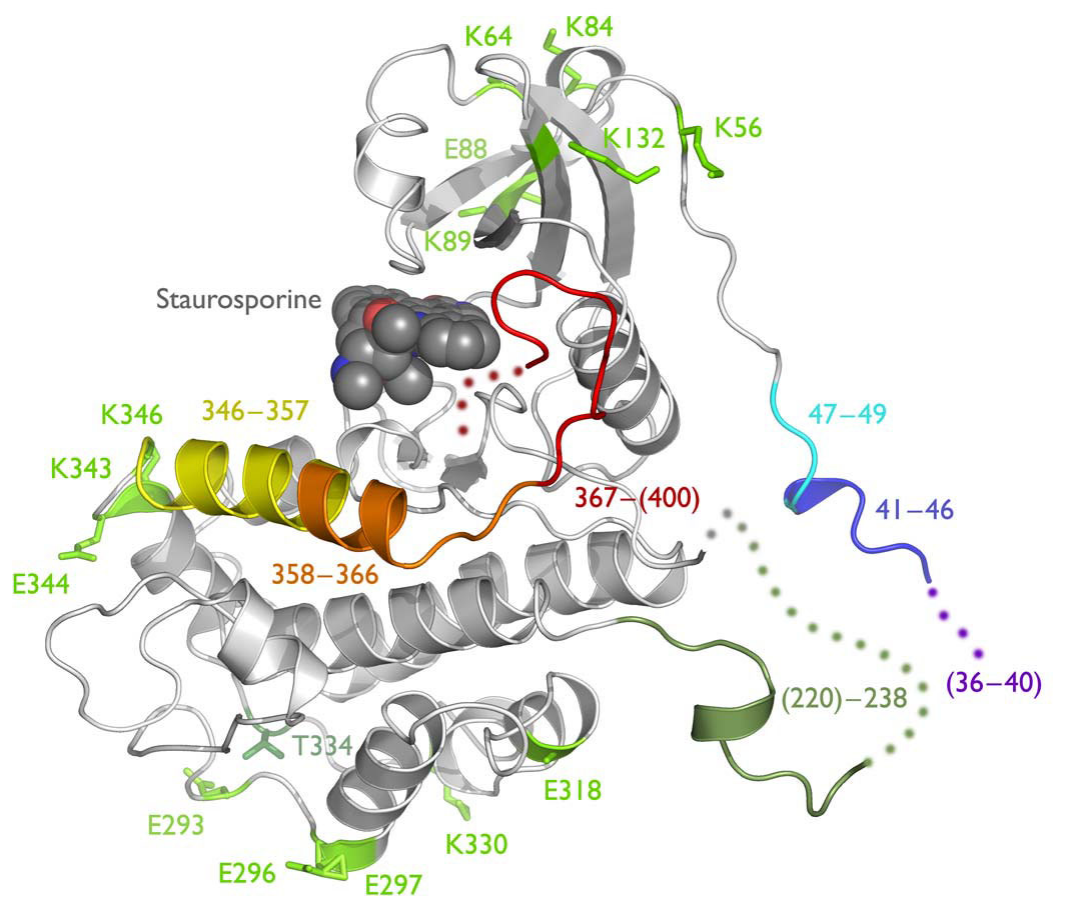
**MK2 structure and location of mutagenesis sites**. MK2 is represented as a ribbon diagram, a composite of Protein Data Bank entries 1KWP and 1NXK. The mutagenesis sites (see Table 1 and Additional File 1 Table S7) are colored by tactic: (1) *N*-terminal variations, purple/blue/cyan, C-terminal variations, yellow/orange/red; (2) entropy-reducing surface mutations, light-green; (3) internal deletions, grey-green; and (4) pseudoactivation mutations, dark-green. Missing residues are represented as dots.

We thus implemented a broad, four-point MK2 construct design strategy coupled with the high-throughput production and testing of multiple crystallographic constructs. First, we sought to parlay the domain organization of MK2 into a series of *N*- and *C*-terminal truncation mutants that either incorporated multiple domains of the intact protein, or defined the kinase domain more precisely than a single construct would. Our approach was significantly more extensive than that of Malawski *et al. *[18]. Second, we mutated surface-exposed lysine and glutamate residues to alanine, to reduce the high conformational entropy of these residues [19]. This approach has been used successfully in a variety of contexts [20]. Third, the flexible internal activation loop of MK2 was deleted. Fourth, phosphorylation sites were altered to provide homogenous MK2 rather than a heterogeneous mixture of unactivated (no phosphorylation) and activated (partial or full phosphorylation) forms. A representative subset of the constructs we used is shown in Table 1; the complete list of constructs is also available (see Additional File 1 Table S7). Figure 1 illustrates the location of all mutation sites mapped onto the MK2 three-dimensional structure.

Our initial construct design tactic was alteration of the *N*- or *C*-termini of full-length MK2, thereby either removing flexible terminal segments that might hinder crystallization or simply providing different termini that might enable different crystal forms (via altered packing, isoelectric point, hydrophobicity, etc.). This approach has been used previously by several groups, and limited systematic *N*- and *C*-terminal truncations have been explored [18]; the construct used most often has been MK2(41–364), a form of the enzyme noted to be constitutively-active [8]. Given the poor behavior of the early constructs that included (part of) the proline-rich *N*-terminal domain, we deleted this domain in all subsequent constructs. We explored several termini: Gln41, His47, and Arg50; and Gln327, Leu342, Asp345, Thr357, Arg364, Asp366, and His400 (Figure 1). These residues delineate the kinase domain at the *N*-terminus, and the kinase domain, the autoinhibitory α-helix, and the NES/NLS at the *C*-terminus.

Our second tactic addressed the protein physical characteristic of surface entropy. Following Longenecker and colleagues [19], a series of point mutants was designed to alter flexible surface lysine and glutamate residues to minimize crystal protein entropy and entropic loss on crystallization. This approach was successfully used with RhoGD1, for which new crystal forms were identified that exhibited enhanced diffraction. Several MK2 mutants in which alanine was substituted for lysine or glutamate are shown in Table 1 (see also Figure 1 and Additional File 1 Table S7). All mutants were constructed at one time, without iterative improvements.

Our third tactic addressed the internal flexibility of MK2. In several of the prior MK2 structures, the kinase activation loop (residues 220–238) engages in significant crystal contacts. We hypothesized that deletion of this long, flexible loop (Figure 1) might drive formation of alternate, better-diffracting crystal lattices. We thus examined two activation loop deletions: MK2(41–364, ΔL220-G238) and MK2(47–366, ΔL220-G238).

Our final tactic sought to reduce the chemical and conformational heterogeneity of MK2. All reported MK2 structures are of the unphosphorylated enzyme. Previous studies had shown that mutation of phosphothreonine residues to glutamate led to constitutive activation of the kinase [16,17]. We reasoned that by altering the activation state of the protein, we would not only access a more homogeneous enzyme but also distinct conformational states, and hence increase the likelihood of useful crystal forms. The pseudoactivated mutants were based on two truncated constructs, MK2(41–364) and MK2(47–366). Glutamate mutations T222E and T334E (both singly, and as the double mutant) replaced the threonines reported to activate MK2 when phosphorylated. We prioritized mutation of T222 and T334 over S272 because existing data suggested that the threonine residues were the more significant MK2 phosphorylation sites [16]. T334 is shown in Figure 1; T222 is located within the disordered portion of the activation loop.

### Expression, Purification and Crystallization

**Figure 2:**
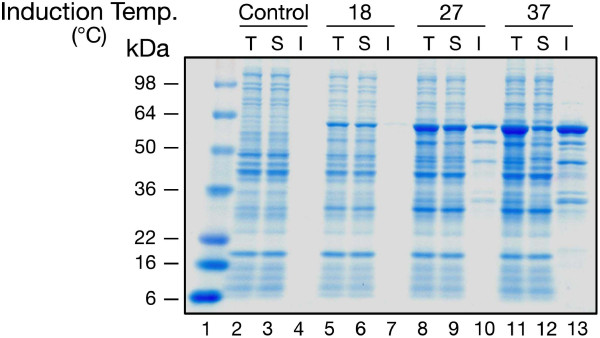
**Analysis of MK2(47–366, T222E) expression as a function of temperature**. Total, soluble, and insoluble fractions (see Methods) were separated by 4–20% SDS-PAGE; stained with Coomassie Blue. Lanes: (1) MW markers; (2–4) control (uninduced); (5–7) 18°C; (8–10) 27°C; (11–13) 37°C;. T, total; S, soluble; I, insoluble. All samples were taken 5 h after induction with IPTG. See also Table 3.

The MK2 constructs (Table 1; Additional File 1 Table S7) were cloned using standard techniques and expressed in *E. coli *as glutathione *S*-transferase (GST) fusion proteins. The expression plasmids encoded GST followed by thrombin and tobacco etch virus (TEV) protease cleavage sites and the desired MK2 sequence. It proved important to develop a method for rapid generation and screening of multiple constructs in parallel. After plasmid construction (PCR, mutagenesis, ligation, etc.) and transformation, typically in parallel sets of 4–8 constructs, test expression was carried out on a small scale, to examine both yield and especially protein solubility. Representative results are shown in Figure 2 and Table 2. Low temperature induction (18°C) provided the optimal balance between expression yield and solubility for most constructs; higher temperatures increased the proportion of insoluble protein. One pseudoactivated construct, MK2(47–366, T222E), exhibited robust expression with both low and medium temperature induction; typical results are also shown in Figure 2. This systematic expression/solubility triage was used for all constructs. Constructs that expressed at high (soluble) levels were prioritized for small-scale purification.

**Table 2:** Solubility assessment for representative MK2 constructs.

**MK2 Construct**		**Solubility**		
**Backbone**	**Mutation(s)**	**18°C**	**27°C**	**37°C**

41–364	--	Low	Low	Low
41–364	K64A	Low	Low	Low
41–364	Δ(L220-G236)	High	High	High
41–364	T222E	Low	Low	Low
41–364	K330A	Low	Low	Low
41–364	T334E	Low	Low	Low
41–364	K343A, K344A, K364A	Low	Low	Low
47–366	--	High	High	Medium
47–366	K64A	High	High	Medium
47–366	K84A	High	Med	Low
47–366	Δ(L220-G236)	High	High	High
47–366	T222E	High	High	Low
47–366	T222E, T334E	Low	Low	Low
47–366	T334E	Low	Low	Low

Expression tests were conducted at three temperatures for each construct. Low (<10%), medium (10–50%) and high (>50%) grades were assigned to each construct based on the ratio of soluble/insoluble MK2 as assessed by SDS-PAGE. An example gel is shown in Figure 2.

**Figure 3:**
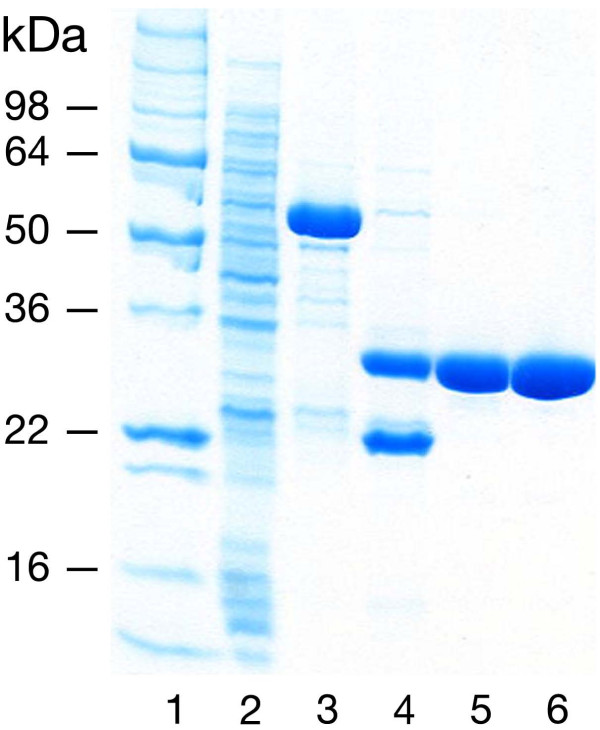
**Representative MK2 purification: MK2(47–366, T222E)**. Protein fractions were separated by 4–20% SDS-PAGE; stained with Coomassie Blue. Lanes: (1) MW markers; (2) glutathione affinity column flow-thru; (3) glutathione affinity column eluate (40 mM glutathione, pH 8.0); (4) TEV protease cleavage; GST is present below MK2; (5) MonoS 10/10 eluate (~200 mM NaCl); (6) Superdex 75 10/60 peak fraction.

Routine procedures (glutathione affinity chromatography, TEV protease cleavage, cation exchange and finally size exclusion chromatography) were used to purify the MK2 constructs. Initially, limited attempts were made to purify proteins using parallel 24-well methods (filtration plates, etc.). But, the rapidity with which conventional purification could be performed made the use of small-scale plate methods unnecessary. Yields from the glutathione affinity chromatography capture step were 4–30 mg/L of culture; the parental constructs MK2(41–364) and MK2(47–366) had crude yields of 5 mg/L. Final yields for all constructs were 0.4–12 mg/L. Constructs that gave the highest yields were progressed first to large-scale purification and crystallization trials; only one construct, MK2(47–366, K56A), produced less than 0.5 mg/L and therefore was not progressed. Example yields for several pseudoactivated constructs are shown in Table 3. MK2(47–366, T222E) was found to be devoid of enzymatic activity, in agreement with previous reports that more than one (pseudo)phosphorylation is required to activate MK2 [16]. A typical example of the high purity afforded by our purification scheme is shown in Figure 3 for MK2(47–366, T222E).

**Table 3:** Summary of pseudoactivated MK2 construct expression, enzymatic activity, and crystallization.

**Construct**	**Yield (mg/L)**		**Enzymatic Activity (cts/nM)**		**Crystallography**	
	
	**Crude**	**Final**	**Without p38 activation**	**With p38 activation**	**Crystals Obtained?**	**Diffraction?**
MK2(41–364, T222E)	3.0	1.0	0.30	0.34	Yes	No
MK2(47–366, T222E)	6.0	4.0	<0.02	<0.02	Yes	Yes
MK2(47–366, T334E)	4.5	2.0	1.5	1.2	Yes	No

**Figure 4:**
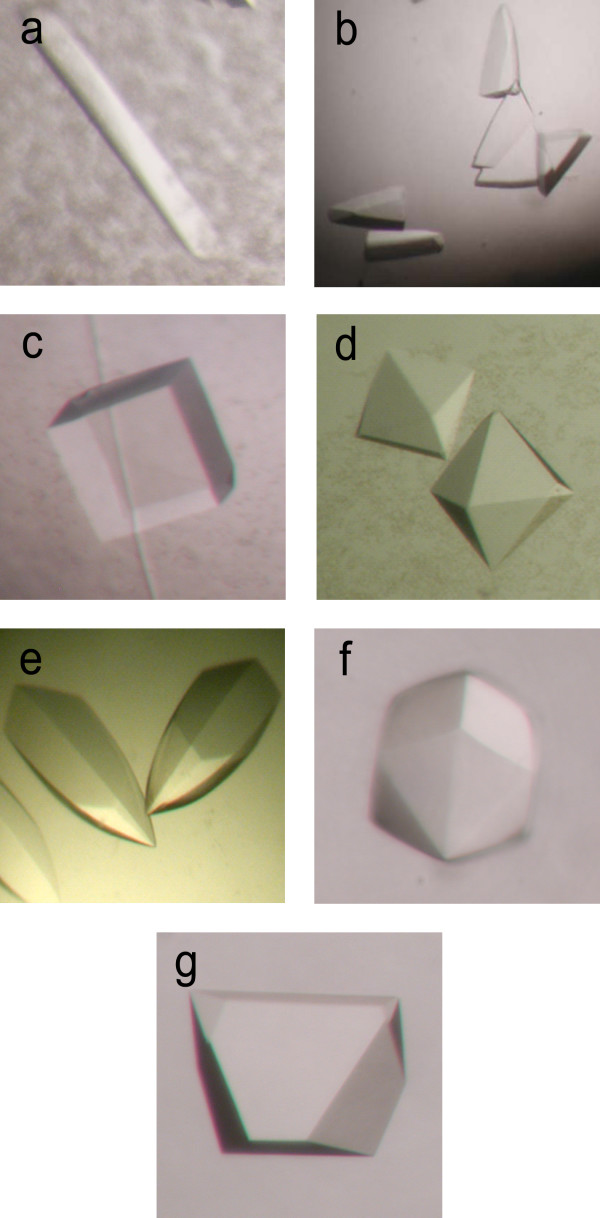
**Seven MK2 crystal forms were identified (Table 5)**. (a) Form I: MK2(41–364, K343A, K344A, K364A); 2 M (NH_4_)_2_SO_4_, 35 mM Cymal^®^-3 (3-cyclohexyl-1-propyl-β-D-maltoside). (b) Form II: MK2(47–366); 2 M (NH_4_)_2_SO_4_, 100 mM HEPES, pH 7.0, 100 mM Li_2_SO_4_. (c) Form III: MK2(47–366); 2 M (NH_4_)_2_SO_4_, 0.1 M Na citrate, pH 5.0, 4% 1,4-butanediol. (d) Form IV: MK2(47–366, T222E), 2 M sodium malonate, pH 5.5, 0.01 mM Anapoe 80. (e) Form V: MK2(41–364); 1.5 M Na malonate, pH 8.0. (f) Form VI: MK2(41–364); 1.8 M Na malonate, pH 8.0. (g) Form VII: MK2(41–364); 1.75 M (NH_4_)_2_SO_4_, 0.1 M Na citrate, pH 8.0. Crystals grew to a maximal size of ~0.1–0.2 × 0.1–0.2 × 0.2 mm (Forms V-VII); other crystals were ~0.1 × 0.1 × 0.1 mm (Forms III & IV) or smaller. See Table 5 for additional details.

Forty-three of forty-four constructs produced crystals using commercial crystallization screens (Hampton Crystal Screen I/II™ and Wizard Screen I/II™). The most common crystallization condition was 2 M (NH_4_)_2_SO_4_, 0.2 M Li_2_SO_4_, 0.1 M CAPS, pH 10.5. Crystals were optimized with an emphasis on finding novel conditions. We created a customized in-house crystallization grid to optimize co-crystallization conditions for a variety of MK2/inhibitor complexes (Table 4; complete crystallization reagent compositions are listed in Additional File 1 Table S8). Seven crystal forms were ultimately identified (Figure 4, Table 5); three of these have been independently identified by other investigators: Form IV [8-10,21]; Form V [9,22]; and Form VII [9]. Beyond MK2, we have since used this and similar grids to crystallize other kinase/inhibitor complexes.

**Table 4:** Customized MK2 robotic crystallization screen conditions.

**Variable**	**Parameters**
Buffer	Citrate HEPES

pH	7.0 – 8.5

Precipitant	Ammonium Sulfate Ammonium Malonate Na Malonate K Malonate Na/K Phosphate

Additives	DMSO 2-Propanol PEG 400 PEG 3350 Na Malonate Na Citrate

The complete screen composition is available in Additional File 1 Table S8.

**Table 5:** Seven MK2 crystal forms.

**Form**	**Construct(s)**	Crystallization Conditions *	**Space Group**	**Unit Cell (*a*, >*b*, *c*, Å)**	**Res. (Å) *N *†**	**Notes**
I	MK2(41–364) & MK2(47–366) surface mutants ‡	AS, LS, & NP pH 5–8	N.D.	N.D.	>11 *N.D.*	Rods; high mosaicity

II	MK2(41–364) & MK2(47–366) surface mutants ‡	AS, LS, & NM pH 5–8	N.D.	N.D.	>11 *N.D.*	Plates; high mosaicity

III	MK2(47–366)	2 M AS 0.1 M Na citrate pH 5.0 4% 1,4-butanediol	*P*2_1_3	215	3.4 *4*	Cubes

IV	MK2(47–366, T222E)	2 M NM pH 5.5 10 μM Anapoe 80	*F*4_1_32	254	2.9 *1*	Bipyramids; inhibitor soaking req'd

V	MK2(41–364)	1.5–1.8 M NM pH 8.0	*P*6_3_	158 158 138	2.6–3.3 *4*	Hex. bullets; co-crystals

VI	MK2(41–364)	1.5–1.8 M NM pH 8.0	*P*6_3_	144 144 152	2.6–3.3 *4*	Hex. bullets; co-crystals; "collapsed" Form V

VII	MK2(41–364)	1.75 M AS 0.1 M Na citrate pH 8.0	*P*2_1_2_1_2_1_	140 180 215	2.6–3.3 *12*	Sharp blocks; co-crystals

* AS – (NH_4_)_2_SO_4_; LS – Li_2_SO_4_; NM – sodium malonate; NP – sodium phosphate.

† Resolution of diffraction. *N *– MK2 molecules/crystallographic asymmetric unit.

‡ Constructs 23, 24, 26, 27, 29, 32, 33, 35, and 36 (Form I), and 9, 24, and 33 (Form II) in Additional File 1 Table S7.

Three pseudoactivated constructs were analyzed for diffraction: MK2(41–364, T222E), MK2(47–366, T222E), and MK2(47–366, T334E) (Table 3). Although all yielded moderate amounts of protein and crystals suitable for diffraction testing, only one, MK2(47–366, T222E), diffracted well. The detergent Anapoe 80 proved to be extremely effective with these crystals, improving diffraction to 2.9-Å resolution (Form IV, Table 6). Crucially, this crystal form was used to solve our first MK2 structure in complex with a prototypical inhibitor chosen from our high-throughput screening lead chemotype. As the project progressed, however, it was supplanted by other crystal forms (especially Forms V-VII) that were more amenable to co-crystallization.

**Table 6:** Crystallographic statistics for an MK2/lead compound inhibitor complex.

**Parameter**	**Value**
Construct	MK2(47–366, T222E)
Crystal form	IV
Space group	*F*4_1_32
Resolution (Å)	20.0–2.9
Unit cell length (*a*; Å)	253.508
Unique reflections	29,178
R_sym_	0.069
⟨ *I/σ*_*I*_ ⟩	10.9
Data completeness (%)	99.7
Mean multiplicity	8.2
*R*_cryst _(%)	22.9
*R*_free _(%)	26.8

### Pseudoactivated MK2 adopts the conformation of inactive MK2

**Figure 5:**
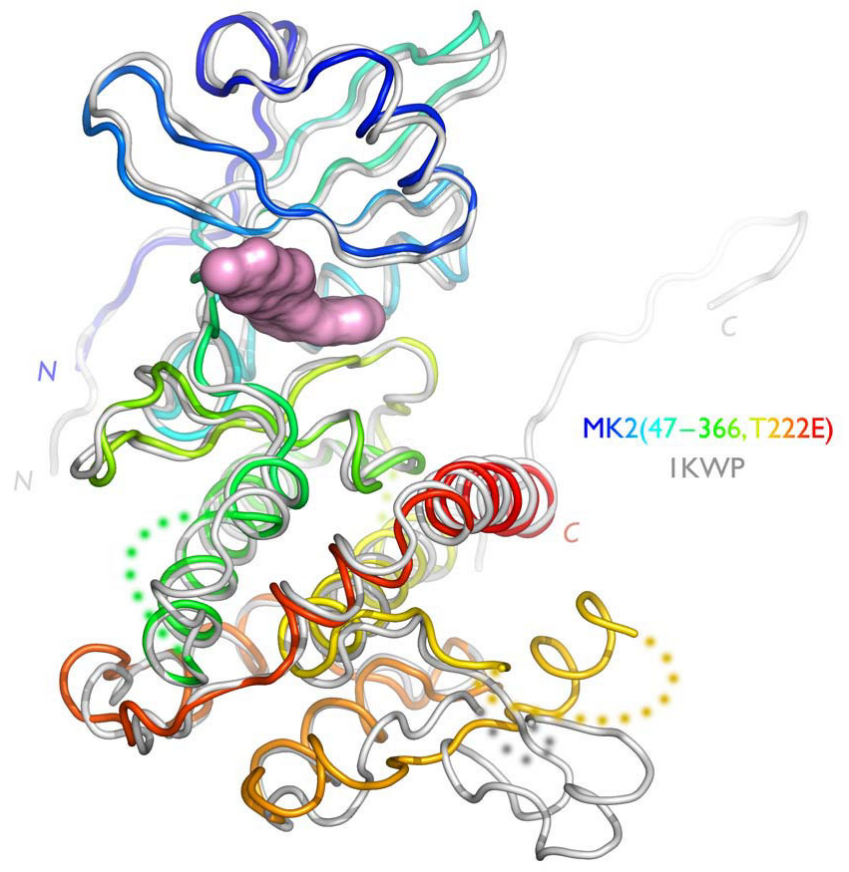
**Structure of MK2(47–366, T222E) bound to a lead compound**. The micromolar-potency inhibitor was soaked into a Form IV crystal. The inhibitor, represented as a tan molecular surface, binds deeply in the ATP pocket of MK2(47–366, T222E) (rainbow coloring). Apo-MK2 (PDB entry 1KWP; light-grey) is shown for comparison.

The crystal structure of pseudoactivated MK2 in complex with a micromolar lead compound was determined in space group *F*4_1_32 (Table 6). This crystal form was reported subsequent to our work by other investigators [8-10]. Superimposition with apo-MK2 (Protein Data Bank entry 1KWP[7]) illustrates a similar "closed" conformation Figure 5. One significant difference is observed in the arrangement of the glycine-rich loop, which assumes a non-canonical orientation by flipping away from the active site to form a short helix. This rearrangement increases the solvent exposure of the ATP binding pocket, making this crystal form a good candidate for inhibitor soaking. We successfully soaked an MK2-specific inhibitor into the ATP binding pocket, leading to our reference crystal structure (Figure 5). Due to the disorder of the activation loop, we were unable to resolve the T222E pseudoactivating mutation. Although the mutation was not directly involved in driving a novel crystal form, through altered crystal packing or (apparently) a significantly altered activation loop conformation, we believe that the enhanced solubility and stability MK2(47–366, T222E) facilitated crystallization.

### Structural Correlates

For reasons that are still unclear, MK2 has an overwhelming propensity to make specific, trimer-forming intermolecular contacts as it crystallizes. Some of our mutations abrogated these contacts; instead of shifting crystallization to new conditions and crystal forms, however, those MK2 constructs simply did not crystallize. Thus, a level of mutagenesis that would be sufficient for most proteins of this size was surprisingly less effective with MK2.

What are these trimers? As noted by Hillig *et al. *[18], two distinct packing interactions are present in both Form IV (space group *F*4_1_32; PDB entry 2JBO[9]) and Form VII (*P*2_1_2_1_2_1_; PDB entry 2JBP[9]) MK2 crystals (Table 5). The Type 1 trimer is mediated by a draping of the *N*-terminus, beginning around residue 47, over the *N*-lobe of another MK2 subunit. Constructs beginning at residues 41 or 47 retain this contact and crystallize; those beginning at residue 50 lose the contact and do not form crystals. The Type 2 trimer is mediated by the *C*-terminal portion of the activation loop packing against helices F, G, and H. Constructs in which the activation loop was deleted, being unable to form these contacts, do not crystallize. Notably, Glu233-Arg313 and Glu238-Arg280 salt bridges mediate Type 2 contacts. Targeting of these glutamate residues in a second round of entropy-reduction mutagenesis might have altered the Type 2 contacts enough to spur formation of other crystal forms.

MK2 trimer formation is due entirely either to crystallographic symmetry (Form IV) or to non-crystallographic symmetry (Form VII). Form IV has one molecule/asymmetric unit, and the two types of trimers are formed by adjacent, non-intersecting crystallographic 3-fold symmetry axes. Conversely, since space group *P*2_1_2_1_2_1 _has no 3-fold axes, the 12 molecules/asymmetric unit in Form VII are arranged such that both trimer types are formed by two non-crystallographic 3-fold axes that nearly intersect in the center of the 12-subunit, virus-like MK2 shell.

Amazingly, *all *characterized MK2 crystal forms shown in Table 5 are composed of Type 1 and/or Type 2 trimers. Forms V and VI (both *P*6_3_; e.g., PDB entry 1NXK[8]) have four molecules/asymmetric unit; three subunits form the Type 1 non-crystallographic trimer; the fourth, "odd man out" subunit forms, through crystallographic symmetry, the Type 2 trimer. And, the tenuous packing in Form III (*P*2_1_3; four molecules/asymmetric unit, 78% solvent) is mediated by trimer formation at two adjacent, non-intersecting crystallographic 3-fold axes, as in the other cubic (Form IV) crystal form. Only the first reported MK2 structure (*R*3; PDB entry 1KWP; two molecules/asymmetric unit) breaks the pattern [7]. Uniquely compared to all other MK2 crystals, the construct used in that study included the complete MK2 *C*-terminus. Packing in this crystal form is mediated by the Type 1 trimer (crystallographic 3-fold) and a novel (parallel, crystallographic) trimeric contact centered at residue 370 that positions the extended C-terminus (ordered to residue ~385) to pack against another MK2 subunit.

Although the biological relevance of MK2 trimer formation is unknown, we note that trimer formation is structurally incompatible with formation of the MK2-p38 complex [23,24]. Thus, MK2 trimer formation may be a form of self-regulation relevant *in vivo*.

In summary, much of the mutagenesis work reported here was, in the end, stymied by the unusually strong proclivity of MK2 toward trimer formation. Nonetheless, our systematic approach did indicate that truncations rather than mutations were more effective for MK2 crystallization. The exact position of the MK2 *N*-terminus is more important than is the *C*-terminus, and the activation loop could not be deleted. Surface arginine and glutamate residues drive crystallization more strongly than do surface lysine residues prevent it. The pseudoactivation constructs were helpful, likely due to slight modulation of protein surface properties rather than by producing a different protein conformation. These unusual MK2 properties result in many closely related (though at first glance *apparently *different) crystal forms (Table 5). Additional surface mutants (including untargeted glutamates, and also arginines) would seem to be required to drive MK2 into truly different crystal forms that can be produced under low ionic strength (e.g., polyethylene glycol) conditions rather than in high salt.

## Conclusion

The systematic methods for the design, production and evaluation of MK2 protein constructs presented here allowed us to reproducibly obtain suitable crystals such that structure-based drug design could proceed. Our methods are robust and allowed rapid evaluation of about fifty constructs. This rapidity enabled the scale-up production of selected MK2 constructs in multi-milligram quantities proteins for structural studies. Using rational site-directed mutagenesis and in-house customized crystallization screens (Table 4), we were able to identify several novel crystallization conditions. Combined with variation of other parameters, such as surface side chain entropy and (pseudo)activation state, these high-throughput techniques produced five new MK2 crystal forms, most with improved diffraction characteristics (Table 5). One such crystal form, grown with a MK2 phosphorylation-site pseudoactivation mutant, was used to solve our first MK2/lead inhibitor complex (Figure 5).

Several key lessons were learned from this exercise. First, setting reasonable criteria for construct performance and prioritization is essential to identify constructs suitable for further evaluation and possible scale-up. In the case of MK2, most constructs gave satisfactory performance in expression and purification. Choices were necessary, however; we prioritized higher-yielding, more soluble constructs (Table 2) for scale-up and more extensive crystallization screening. Implicitly, we assumed that constructs that expressed or purified poorly were less likely to crystallize well. Our assumption appears to be supported by the expression yield, protein melting point, and crystallization data of Malawski *et al. *[18]. Second, a systematic crystallization screen (Table 4; see also Additional File 1 Table S8) was required to identify crystallization conditions in a robust manner. Indeed, the interplay between multiple constructs and multiple, customized crystallization solutions likely contributed greatly to our success. We note that the composition of customized crystallization screens is protein-specific – for MK2, we used a preponderance of high ionic strength conditions, since repeated screening with, for example, PEG solutions never provided crystals. Other proteins will behave differently. Third, reduction of surface side chain entropy can require several iterative rounds before productive mutation sites are identified. In retrospect, more than one round of surface residue mutations in this study, optimally informed by initial structural information, might have yielded additional crystal forms (e.g., mutation of Glu233 and Glu238 to disrupt the Type 2 trimer contact). Fourth, high throughput construct exploration must be initiated early in a drug discovery program in order to synchronize with hit-to-lead synthetic chemistry efforts, preferably in concert with initial target selection studies, i.e. *before *a high-throughput inhibitor screen is begun. This head start is especially critical for problematic crystallization targets like MK2. Applying these lessons learned from our experience with MK2 has helped us to accelerate many other structural programs, enabling us to impact lead discovery programs more rapidly and efficiently.

## Methods

### Cloning

Most human MK2  constructs were engineered as fusion proteins with *Schistosoma japonicum *glutathione *S*-transferase (GST; ), using the pGEX4T-1 vector (GE Healthcare). The sequence used was: GST-(SDLVPR_↑_GSENLYFQ_↑_G)-MK2. The linker sequence encodes thrombin and tobacco etch virus (TEV) protease cleavage sites ("↑"). Two protease sites were included for maximal flexibility in removal of the fusion tag after purification; we almost exclusively used the TEV protease site, resulting in an unnatural glycine residue *N*-terminal to MK2. A few constructs were also made as His_6_-FLAG-TEV-MK2 fusion proteins, using the pET21a+ vector (Invitrogen). The sequence used was: (MGHHHHHHGSGDYKDDDDKDYDIPTTENLYFQ_↑_G)-MK2. We refer to these vectors as "pGEX4T-1-GST-Thr-TEV" and "pET21a+-His_6_-FLAG-TEV", respectively.

Mutagenic primers were designed according to the QuikChange XL Site-Directed Mutagenesis Kit (Stratagene) instructions and were purchased from Invitrogen. Briefly, primer pairs were made for each mutation. Both primers in each pair contained the desired mutation and annealed to the same sequences on opposite strands of the plasmid template. The desired mutations (nucleotide replacements or deletions), in the middle of each primer, were flanked by 15–19 bases of wildtype sequence on both sides. Primer stocks and dilutions were arrayed in 96-well V-bottom microplates (MJ Research) and stored at -20°C.

Site-directed mutagenesis was performed using the QuikChange kit (Stratagene) using the plasmids pGEX4T-1-GST-Thr-TEV-MK2(47–366) and pGEX4T-1-GST-Thr-TEV-MK2(41–364) as templates. PCR was performed in 96-well V-bottom microplates using a DNA Engine Dyad (MJ Research) thermocycler complete with an ALS-1296, 96-well alpha unit (MJ Research). Reaction mixtures were cycled 18 times according to this schedule: 95°C, 50 s; 60°C, 50 s; 68°C, 14 min. Cycling was preceded by incubation at 95°C (1 min) and followed by incubation at 68°C (7 min).

Transformation of XL-10 Gold Ultracompetent *E. coli *cells (Stratagene) was performed in 24-well RB Blocks (Qiagen). Thawed cells (45 μL) were mixed on ice with 2 μL of 2-mercaptoethanol in each well. A 5 μL aliquot of each PCR reaction mix was added to the appropriate well and the mixture was incubated on ice for 10 min. The blocks were then heat-shocked by immersing the lower half of the blocks into a 42°C water bath (30 s), then placing the block back on ice (2 min). SOC medium (0.5 mL; preheated to 42°C) was then added to each well and the block was immediately placed in an incubator at 37°C for 1 hr with shaking. A 0.1 mL aliquot of each transformation was spread onto 100 mm LB agar plates containing 100 μg/mL ampicillin and incubated overnight at 37°C.

Plasmid DNA was prepared using the QIAprep 8 Turbo Miniprep Kit (Qiagen) in combination with the Qiavac 6S vacuum manifold (Qiagen) according to manufacturer's instructions. The DNA was quantitated spectrophotometrically and diluted to 100 μg/mL with water for sequence analysis. The coding sequence of all constructs was verified.

### Expression

Plasmids encoding the MK2 constructs were transformed into BL21 (DE3) strain for expression studies. Competent cells were transformed with 0.5 μL of each plasmid in 24-well blocks as above (without 2-mercaptoethanol). The transformation mix (0.1 mL) was spread onto 100 mm LB agar plates containing 100 μg/mL ampicillin and incubated overnight at 37°C. Starter cultures grown at 37°C were used to inoculate 2.5 mL of LB medium containing 100 μg/mL ampicillin in 24 deep-well blocks. Once an OD_600 _of 0.4–0.6 was reached, the blocks were shifted to 18°C for 1 h and then induced with IPTG (0.4 mM) for 4 h. Cells were harvested by centrifugation, frozen at -80°C. Large-scale cultures were induced in the same way with the exception that the flasks were shifted to 4°C for 40 min prior to induction.

Analysis of the soluble and insoluble fractions was performed by SDS-PAGE on 4–20% gels (Invitrogen). Cells were thawed and resuspended for lysis in Bug Buster HT (Novagen; 0.25 mL per unit OD_600_). Bugbuster HT containing 60 kU rLysozyme (Novagen; 0.1 mL) was added to the cell suspension (0.1 mL). The mixture was incubated on ice for 15 min before addition of 0.2 mL of Bugbuster HT containing 20 mM DTT and Complete, EDTA-free Protease Inhibitor cocktail (Roche; 1 tablet/30 mL Bugbuster HT). An aliquot of the crude lysate was separated by centrifugation; the supernatant was analyzed as the soluble fraction. The pellet was resuspended in an equal volume of BugBuster HT and analyzed as the insoluble fraction. Proteins were visualized with SimplyBlue stain (Invitrogen)

Western blotting was used to confirm protein identity. Gels were transferred to PVDF membranes using a Protean II Mini Trans-Blot apparatus (BioRad). After blocking membranes with 5% nonfat dry milk in TBS-T buffer (50 mM Tris·HCl, pH 8.0, 150 mM NaCl, 0.1% Tween-20), the blot was probed with horseradish peroxidase-conjugated anti-GST antibody diluted 1:1000 in TBS-T/5% nonfat milk. Membranes were washed extensively with TBS-T, and then bound antibodies were visualized using the SuperSignal West Pico chemiluminescent substrate kit (Pierce).

### Purification

MK2 variants were purified using the following procedure at 4°C: Cell pellets were thawed and resuspended in lysis buffer containing 50 mM Tris·HCl, 250 mM NaCl, 10% glycerol, 1 mM DTT, 1 mM EDTA, pH 7.5. The cell suspension was sonicated on ice for 20 s iterations and then centrifuged (22,000 × *g*) for 45 min. A 10 mL glutathione affinity column (GE Healthcare) was prepared by washing with ten column volumes of Buffer A (50 mM Tris·HCl, 250 mM NaCl, 10% glycerol, 2.5 mM DTT, 1 mM EDTA, pH 8.0) containing Complete, EDTA-free Protease Inhibitor cocktail. Soluble cell lysate was applied to the column and then extensively washed with Buffer A. MK2 was eluted from the column with Buffer A + 40 mM glutathione. The GST tag was cleaved using TEV protease, typically 4–16 h at 4–15°C. The sample was then diluted ten-fold with Buffer B (50 mM MES, 10% glycerol, pH 6.0) and loaded onto a MonoS 10/10 column (GE Healthcare) equilibrated with Buffer B + 20 mM NaCl. The protein was eluted at ≈200 mM NaCl using a stepwise Buffer B/NaCl gradient. MK2-containing fractions were pooled and concentrated to 10 mg/mL, and then loaded onto a Superdex 75 16/60 column (GE Healthcare) equilibrated with 50 mM Tris·HCl, 250 mM NaCl, 10% glycerol, 1 mM DTT, pH 7.5; protein was eluted at 1 mL/min. MK2-containing fractions were pooled and sample purity was assessed by SDS-PAGE; protein identity was confirmed using mass spectrometry.

### Activity Assays

Enzymatic assays utilized a homogeneous time-resolved fluorescence method (CisBio-US, Inc.) to quantitate product formation. Reactions contained in 40 μL: varying amounts of enzyme, 4 μM peptide substrate (Biotin-Ahx-AKVSRSGLYRSPSMPENLNRPR), 10 μM ATP, 20 mM MOPS, 10 mM MgCl_2_, 5 mM EGTA, 5 mM 2-phosphoglycerol, 1 mM Na_3_VO_4_, 0.01% Triton X-100, 5% DMSO, 1 mM DTT, pH 7.2. After 60 min at room temperature, reactions were quenched by adding 10 μL of 0.5 M EDTA. Phosphorylated peptide was measured by addition of 75 μL of 24 ng/mL Eu^3+^-cryptate-labeled anti-phospho-14-3-3 binding motif (CisBio-US, Inc.), 1.47 μg/mL SureLight™ allophycocyanin-streptavidin (CisBio-US, Inc.), 50 mM HEPES, 0.1% BSA, 0.01% Tween-20, 0.4 M KF. The developed reaction was incubated in the dark at room temperature for 10 min, then read in a time-resolved fluorescence detector (Perkin Elmer Discovery or BMG Rubystar) at 620 nm and 665 nm simultaneously, using a 337 nm nitrogen laser for excitation. The 665/620 emission ratio is proportional to the amount of phosphorylated peptide product. Since the HTRF method does not provide absolute quantities of product formed, specific activities were calculated as HTRF counts/nM MK2 protein.

### Crystallization

MK2 constructs yielding more than 1 mg/L of culture were progressed to crystallization trials. Protein was concentrated to 10 mg/mL using an Ultrafree-15 Biomax 10 kDa molecular weight cut-off centrifugal filter device (Millipore). Various inhibitors were added individually to concentrated protein stocks to a final concentration of 1 mM. Complexed MK2 protein (0.5 μL) was mixed with 0.5 μL of various crystallization solutions from Crystal Screen 1™ and Crystal Screen 2™ (Hampton Research) and Wizard Screen 1™ and Wizard Screen 2™ (DeCode Biostructures). The resulting drops were dispensed into 96-well sitting drop trays (Greiner) using a Hydra II+1 liquid handler (Thermo Scientific Matrix). Trays were stored at 18°C and visualized manually. Crystallization was tested extensively at 4°C, uniformly without success.

Accumulated MK2 crystallization hits suggested parameters to be explored more closely in a customized robotic screen, the most effective being the precipitating reagent and pH range. A complete 96-well screen, designed for use with the Hydra II+1, consisted of four 4 × 6 grid screens: (1) (NH_4_)_2_SO_4_/sodium citrate, pH 7–8.5; (2) sodium malonate, pH 7–8.5; (3) sodium phosphate; pH 7–8.5; and (4) a randomized screen obtained by randomly mixing the above three precipitants with other additives (Table 4; see also Additional File 1 Table S8). Seven different crystal forms were identified from this comprehensive screen, as shown in Table 5 and Figure 4.

Crystals for inhibitor soaking were grown in sitting drops by the vapor-diffusion method using MK2(47–366, T222E). MK2 (1.5 μL, 10 mg/mL) was added to 1.5 μL of reservoir solution (2 M sodium malonate, pH 5.5, and 0.01 mM Anapoe 80 [Hampton Research]) and then the drop was sealed in vapor contact with 500 μL of reservoir solution. Crystals grew to about 0.2 mm in size in 3 days. For soaking, one MK2 crystal was added to 60 μL of 1 mM inhibitor dissolved in mother liquor and incubated at 18°C overnight.

### Diffraction Testing and Structure Determination

MK2/inhibitor complex crystals were harvested into a cryoprotectant solution (20% glycerol plus mother liquor) using a fiber loop and flash-cooled in liquid nitrogen. Cystals were stored in liquid nitrogen until diffraction testing. X-ray diffraction testing was conducted in-house using a FR591 rotating anode generator (Bruker AXS) with a MAR345 image plate detector (MARResearch) and Osmic optics (Rigaku USA). A total of 535 crystals were tested, and over 80 crystals were selected for synchrotron data collection if diffraction reached at least 3.5-Å resolution. Advanced Photon Source (Argonne, IL) and National Synchrotron Light Source (Upton, NY) synchrotron beamlines were used primarily for data collection, although a few crystals were selected for in-house data collection.

Diffraction data was processed with the HKL2000 program suite [25]. After determining the crystal orientation, the data were integrated with DENZO, scaled and merged with SCALEPACK, and placed on an absolute scale and reduced to structure factor amplitudes with TRUNCATE [26]. Five percent of the unique reflections were assigned, in a random fashion, to the "free" set, for calculation of the free *R*-factor (*R*_free_) [27]; the remaining 95% of reflections constituted the "working" set, for calculation of the *R*-factor (*R*_cryst_). The x-ray diffraction data for a representative inhibitor-soaked MK2 crystal (Form IV) are summarized in Table 6.

The CCP4 program suite was used to solve and refine the structure [28]. The cross-rotation function was calculated using MOLREP [29], using the apo MK2 structure reported previously (Protein Data Bank entry 1KWP; [7]) as the search model. Initial coordinates were generated based on the one solution apparent at 2.9 Å resolution. Refinement began with rigid-body refinement in REFMAC [30], resulting in an *R*_cryst _of 37.0% (*R*_free _40.0%) for all reflections with |*F*| > 2.0σ_*F*_, 20–2.9 Å. Manual rebuilding of the model was conducted using the molecular graphics program O [31] and examination of sigmaA-weighted 2*F*_O_-*F*_C _and *F*_O_-*F*_C _electron-density maps [32]. Restrained refinement using REFMAC converged at an *R*_cryst _of 22.9% (*R*_free _26.8%), 20–2.9 Å. The quality of the model was assessed with PROCHECK [33] and WHATCHECK [34].

## Authors' contributions

MAA led the MK2 structural biology sub-team; additionally, she participated in construct design, provided protein purification and crystallization oversight, collected diffraction data, and performed the structure determination. SS made the majority of the constructs and performed some of the protein expression. DB, DM, TX, JH, and MP purified protein, set up crystallizations, and collected diffraction data. MT and MD contributed protein characterization data. CH and SK contributed enzymatic data. JD and BN performed the bulk of the protein expression. LQ contributed the early template constructs. DWB provided structural biology oversight; additionally, he participated in construct design. JM provided construct construction and protein expression oversight and participated in construct design. RVT led the MK2 drug discovery project and participated in construct design. RS led the MK2 high-throughput construct construction and protein expression sub-team and participated in construct design. MAA, DWB and RS jointly prepared the manuscript, in consultation with all of the co-authors. All authors have read and approved this manuscript.

## Supplementary Material

Additional file 1 **Complete list of MK2 expression constructs and MK2 robotic crystallization screen used for identification of crystallization hits.** Table S7 is a complete listing of MK2 expression constructs with crystal forms identified. Table S8 is a complete matrix listing of the crystallization screen used in the identification of crystallization hits. Click here for file
