# Supplementary material for: Rational mutagenesis to support structure-based drug design: MAPKAP kinase 2 as a case study
Source: BMC Struct Biol. 2009 Mar 18;9:16. doi: 10.1186/1472-6807-9-16 (PMC2678131; doi:10.1186/1472-6807-9-16)
Supplement: Additional file 1 — Complete list of MK2 expression constructs and MK2 robotic crystallization screen used for identification of crystallization hits. Table S7 is a complete listing of MK2 expression constructs with crystal forms identified. Table S8 is a complete matrix listing of the crystallization screen used in the identification of crystallization hits. [file 1472-6807-9-16-S1.doc]

Table S7. Complete list of MK2 expression constructs.

| **Construct** | **Vector** | **Human MK2** | **Tactic *** | **Crystal Form(s)** |
| --- | --- | --- | --- | --- |
| 1 | pGEX4T-1–GST–Thr | 36–400, K93R | – |  |
| 2 | pGEX4T-1–GST–Thr–TEV | 41–342 | 1 |  |
| 3 | pGEX4T-1–GST–Thr–TEV | 41–345 | 1 |  |
| 4 | pGEX4T-1–GST–Thr–TEV | 41–357 | 1 |  |
| 5 | pGEX4T-1–GST–Thr–TEV | 41–364 | 1 | V, VI, VII |
| 6 | pGEX4T-1–GST–Thr–TEV | 47–342 | 1 |  |
| 7 | pGEX4T-1–GST–Thr–TEV | 47–345 | 1 |  |
| 8 | pGEX4T-1–GST–Thr–TEV | 47–357 | 1 |  |
| 9 | pGEX4T-1–GST–Thr–TEV | 47–366 | 1 | II, III |
| 10 | pET21a^+^–His_6_–FLAG–TEV | 47–366 | 1 |  |
| 11 | pGEX4T-1–GST–Thr–TEV | 47–400 | 1 |  |
| 12 | pET21a^+^–His_6_–FLAG–TEV | 47–400 | 1 |  |
| 13 | pGEX4T-1–GST–Thr–TEV | 50–327 | 1 |  |
| 14 | pET21a^+^–His_6_–FLAG–TEV | 50–327 | 1 |  |
| 15 | pGEX4T-1–GST–Thr–TEV | 50–366 | 1 |  |
| 16 | pET21a^+^–His_6_–FLAG–TEV | 50–366 | 1 |  |
| 17 | pGEX4T-1–GST–Thr–TEV | 50–400 | 1 |  |
| 18 | pET21a^+^–His_6_–FLAG–TEV | 50–400 | 1 |  |
|  |  |  |  |  |
| 19 | pGEX4T-1–GST–Thr–TEV | 41–364, K56A | 2 |  |
| 20 | pGEX4T-1–GST–Thr–TEV | 41–364, K64A | 2 |  |
| 21 | pGEX4T-1–GST–Thr–TEV | 41–364, K84A | 2 |  |
| 22 | pGEX4T-1–GST–Thr–TEV | 41–364, E88A, K89A | 2 |  |
| 23 | pGEX4T-1–GST–Thr–TEV | 41–364, K132A | 2 | I |
| 24 | pGEX4T-1–GST–Thr–TEV | 41–364, E293A, E296A, E297A | 2 | I, II |
| 25 | pGEX4T-1–GST–Thr–TEV | 41–364, E318A | 2 |  |
| 26 | pGEX4T-1–GST–Thr–TEV | 41–364, K330A | 2 | I |
| 27 | pGEX4T-1–GST–Thr–TEV | 41–364, E343A, E344A, E346A | 2 | I |
| 28 | pGEX4T-1–GST–Thr–TEV | 47–366, K56A | 2 |  |
| 29 | pGEX4T-1–GST–Thr–TEV | 47–366, K64A | 2 | I |
| 30 | pGEX4T-1–GST–Thr–TEV | 47–366, K84A | 2 |  |
| 31 | pGEX4T-1–GST–Thr–TEV | 47–366, E88A, K89A | 2 |  |
| 32 | pGEX4T-1–GST–Thr–TEV | 47–366, K132A | 2 | I |
| 33 | pGEX4T-1–GST–Thr–TEV | 47–366, E293A, E296A, E297A | 2 | I, II |
| 34 | pGEX4T-1–GST–Thr–TEV | 47–366, K318A | 2 |  |
| 35 | pGEX4T-1–GST–Thr–TEV | 47–366, K330A | 2 | I |
| 36 | pGEX4T-1–GST–Thr–TEV | 47–366, K343A, E344A, K346A | 2 | I |
|  |  |  |  |  |
| 37 | pGEX4T-1–GST–Thr–TEV | 41–364, Δ(L220–G238) | 3 |  |
| 38 | pGEX4T-1–GST–Thr–TEV | 47–366, Δ(L220–G238) | 3 |  |
|  |  |  |  |  |
| 39 | pGEX4T-1–GST–Thr–TEV | 41–364, T222E | 4 |  |
| 40 | pGEX4T-1–GST–Thr–TEV | 41–364, T222E, T334E | 4 |  |
| 41 | pGEX4T-1–GST–Thr–TEV | 41–364, T334E | 4 |  |
| 42 | pGEX4T-1–GST–Thr–TEV | 47–366, T222E | 4 | IV |
| 43 | pGEX4T-1–GST–Thr–TEV | 47–366, T222E, T334E | 4 |  |
| 44 | pGEX4T-1–GST–Thr–TEV | 47–366, T334E | 4 |  |

* Tactics: 1 – *N*- and *C*-terminal variants; 2 – entropy-reducing side chain mutations; 3 –internal deletion mutations; 4 – phosphorylation mutations.

Table S8: MK2 robotic crystallization screen used for identification of crystallization hits.

| **Buffer** | |  | **pH** |  | **Precipitant** | |  | **Additive** | |
| --- | --- | --- | --- | --- | --- | --- | --- | --- | --- |
| **Reagent** | **M** |  |  |  | **Reagent** | **M** |  | **Reagent** | **%** |
| Na Citrate | 0.1 |  | 7.0 |  | Ammonium Sulfate | 1.2 |  | — |  |
| Na Citrate | 0.1 |  | 7.5 |  | Ammonium Sulfate | 1.2 |  | — |  |
| Na Citrate | 0.1 |  | 8.0 |  | Ammonium Sulfate | 1.2 |  | — |  |
| Na Citrate | 0.1 |  | 8.5 |  | Ammonium Sulfate | 1.2 |  | — |  |
| Na Citrate | 0.1 |  | 7.0 |  | Ammonium Sulfate | 1.4 |  | — |  |
| Na Citrate | 0.1 |  | 7.5 |  | Ammonium Sulfate | 1.4 |  | — |  |
| Na Citrate | 0.1 |  | 8.0 |  | Ammonium Sulfate | 1.4 |  | — |  |
| Na Citrate | 0.1 |  | 8.5 |  | Ammonium Sulfate | 1.4 |  | — |  |
| Na Citrate | 0.1 |  | 7.0 |  | Ammonium Sulfate | 1.6 |  | — |  |
| Na Citrate | 0.1 |  | 7.5 |  | Ammonium Sulfate | 1.6 |  | — |  |
| Na Citrate | 0.1 |  | 8.0 |  | Ammonium Sulfate | 1.6 |  | — |  |
| Na Citrate | 0.1 |  | 8.5 |  | Ammonium Sulfate | 1.6 |  | — |  |
| Na Citrate | 0.1 |  | 7.0 |  | Ammonium Sulfate | 1.8 |  | — |  |
| Na Citrate | 0.1 |  | 7.5 |  | Ammonium Sulfate | 1.8 |  | — |  |
| Na Citrate | 0.1 |  | 8.0 |  | Ammonium Sulfate | 1.8 |  | — |  |
| Na Citrate | 0.1 |  | 8.5 |  | Ammonium Sulfate | 1.8 |  | — |  |
| Na Citrate | 0.1 |  | 7.0 |  | Ammonium Sulfate | 2.0 |  | — |  |
| Na Citrate | 0.1 |  | 7.5 |  | Ammonium Sulfate | 2.0 |  | — |  |
| Na Citrate | 0.1 |  | 8.0 |  | Ammonium Sulfate | 2.0 |  | — |  |
| Na Citrate | 0.1 |  | 8.5 |  | Ammonium Sulfate | 2.0 |  | — |  |
| Na Citrate | 0.1 |  | 7.0 |  | Ammonium Sulfate | 2.2 |  | — |  |
| Na Citrate | 0.1 |  | 7.5 |  | Ammonium Sulfate | 2.2 |  | — |  |
| Na Citrate | 0.1 |  | 8.0 |  | Ammonium Sulfate | 2.2 |  | — |  |
| Na Citrate | 0.1 |  | 8.5 |  | Ammonium Sulfate | 2.2 |  | — |  |
| — |  |  | 7.0 |  | Na Malonate | 1.2 |  | — |  |
| — |  |  | 7.5 |  | Na Malonate | 1.2 |  | — |  |
| — |  |  | 8.0 |  | Na Malonate | 1.2 |  | — |  |
| — |  |  | 8.5 |  | Na Malonate | 1.2 |  | — |  |
| — |  |  | 7.0 |  | Na Malonate | 1.4 |  | — |  |
| — |  |  | 7.5 |  | Na Malonate | 1.4 |  | — |  |
| — |  |  | 8.0 |  | Na Malonate | 1.4 |  | — |  |
| — |  |  | 8.5 |  | Na Malonate | 1.4 |  | — |  |
| — |  |  | 7.0 |  | Na Malonate | 1.6 |  | — |  |
| — |  |  | 7.5 |  | Na Malonate | 1.6 |  | — |  |
| — |  |  | 8.0 |  | Na Malonate | 1.6 |  | — |  |
| — |  |  | 8.5 |  | Na Malonate | 1.6 |  | — |  |
| — |  |  | 7.0 |  | Na Malonate | 1.8 |  | — |  |
| — |  |  | 7.5 |  | Na Malonate | 1.8 |  | — |  |
| — |  |  | 8.0 |  | Na Malonate | 1.8 |  | — |  |
| — |  |  | 8.5 |  | Na Malonate | 1.8 |  | — |  |
| — |  |  | 7.0 |  | Na Malonate | 2.0 |  | — |  |
| — |  |  | 7.5 |  | Na Malonate | 2.0 |  | — |  |
| — |  |  | 8.0 |  | Na Malonate | 2.0 |  | — |  |
| — |  |  | 8.5 |  | Na Malonate | 2.0 |  | — |  |
| — |  |  | 7.0 |  | Na Malonate | 2.2 |  | — |  |
| — |  |  | 7.5 |  | Na Malonate | 2.2 |  | — |  |
| — |  |  | 8.0 |  | Na Malonate | 2.2 |  | — |  |
| — |  |  | 8.5 |  | Na Malonate | 2.2 |  | — |  |
| — |  |  | 7.0 |  | Na/K Phosphate | 1.2 |  | — |  |
| — |  |  | 7.5 |  | Na/K Phosphate | 1.2 |  | — |  |
| — |  |  | 8.0 |  | Na/K Phosphate | 1.2 |  | — |  |
| — |  |  | 8.5 |  | Na/K Phosphate | 1.2 |  | — |  |
| — |  |  | 7.0 |  | Na/K Phosphate | 1.4 |  | — |  |
| — |  |  | 7.5 |  | Na/K Phosphate | 1.4 |  | — |  |
| — |  |  | 8.0 |  | Na/K Phosphate | 1.4 |  | — |  |
| — |  |  | 8.5 |  | Na/K Phosphate | 1.4 |  | — |  |
| — |  |  | 7.0 |  | Na/K Phosphate | 1.6 |  | — |  |
| — |  |  | 7.5 |  | Na/K Phosphate | 1.6 |  | — |  |
| — |  |  | 8.0 |  | Na/K Phosphate | 1.6 |  | — |  |
| — |  |  | 8.5 |  | Na/K Phosphate | 1.6 |  | — |  |
| — |  |  | 7.0 |  | Na/K Phosphate | 1.8 |  | — |  |
| — |  |  | 7.5 |  | Na/K Phosphate | 1.8 |  | — |  |
| — |  |  | 8.0 |  | Na/K Phosphate | 1.8 |  | — |  |
| — |  |  | 8.5 |  | Na/K Phosphate | 1.8 |  | — |  |
| — |  |  | 7.0 |  | Na/K Phosphate | 2.0 |  | — |  |
| — |  |  | 7.5 |  | Na/K Phosphate | 2.0 |  | — |  |
| — |  |  | 8.0 |  | Na/K Phosphate | 2.0 |  | — |  |
| — |  |  | 8.5 |  | Na/K Phosphate | 2.0 |  | — |  |
| — |  |  | 7.0 |  | Na/K Phosphate | 2.2 |  | — |  |
| — |  |  | 7.5 |  | Na/K Phosphate | 2.2 |  | — |  |
| — |  |  | 8.0 |  | Na/K Phosphate | 2.2 |  | — |  |
| — |  |  | 8.5 |  | Na/K Phosphate | 2.2 |  | — |  |
| Na HEPES | 0.1 |  | 7.5 |  | Ammonium Sulfate | 2.0 |  | PEG 400 | 2 |
| Na HEPES | 0.1 |  | 7.5 |  | Ammonium Sulfate | 2.0 |  | DMSO | 2 |
| Na HEPES | 0.1 |  | 7.5 |  | Ammonium Sulfate | 2.0 |  | 2-Propanol | 2 |
| Na HEPES | 0.1 |  | 7.5 |  | Ammonium Sulfate | 2.0 |  | PEG 3350 | 2 |
| Na Citrate | 0.1 |  | 7.5 |  | Ammonium Sulfate | 2.0 |  | PEG 400 | 2 |
| Na Citrate | 0.1 |  | 7.5 |  | Ammonium Sulfate | 2.0 |  | DMSO | 2 |
| Na Citrate | 0.1 |  | 7.5 |  | Ammonium Sulfate | 2.0 |  | 2-Propanol | 2 |
| Na Citrate | 0.1 |  | 7.5 |  | Ammonium Sulfate | 2.0 |  | PEG 3350 | 2 |
| — |  |  | 5.5 |  | Na/K Phosphate | 2.0 |  | — |  |
| — |  |  | 6.0 |  | Na/K Phosphate | 2.0 |  | — |  |
| — |  |  | 6.5 |  | Na/K Phosphate | 2.0 |  | — |  |
| — |  |  | 8.0 |  | Na Malonate | 2.0 |  | PEG 400 | 2 |
| — |  |  | 8.0 |  | Na Malonate | 2.0 |  | DMSO | 2 |
| — |  |  | 8.0 |  | Na Malonate | 2.0 |  | 2-Propanol | 2 |
| — |  |  | 8.0 |  | Na Malonate | 2.0 |  | PEG 3350 | 2 |
| — |  |  | 8.0 |  | Na/K Phosphate | 2.0 |  | PEG 400 | 2 |
| — |  |  | 8.0 |  | Na/K Phosphate | 2.0 |  | DMSO | 2 |
| — |  |  | 8.0 |  | Na/K Phosphate | 2.0 |  | 2-Propanol | 2 |
| — |  |  | 8.0 |  | Na/K Phosphate | 2.0 |  | PEG 3350 | 2 |
| — |  |  | 8.0 |  | Ammonium Sulfate | 1.0 |  | Na Malonate | 1.0 M |
| — |  |  | 8.0 |  | Ammonium Sulfate | 1.0 |  | Na Citrate | 0.5 M |
| — |  |  | 8.0 |  | Ammonium Malonate | 2.0 |  | — |  |
| — |  |  | 8.0 |  | K Malonate | 2.0 |  | — |  |
